# The effect of long-term exposure to single and mixed mineral ions related to water hardness on Nile tilapia (*Oreochromis Niloticus*)

**DOI:** 10.1007/s10695-025-01532-9

**Published:** 2025-07-17

**Authors:** Eman I. Hassanen, Wafaa A. Mohamed, Hanan S. Khalefa, Ghada E. Ali, Mahmoud A. Mahmoud

**Affiliations:** 1 Department of Pathology, Faculty of Veterinary Medicine, Cairo University, P.O. Box 12211, Giza, Egypt; 2 Department of Veterinary Hygiene and Management, Faculty of Veterinary Medicine, Cairo University, Giza, 12211 Egypt; 3 Department of Biochemistry and Molecular Biology, Faculty of Veterinary Medicine, Cairo University, Giza, 12211 Egypt

**Keywords:** Hardness, Histopathology, Nile tilapia, Oxidative stress, Toxicity

## Abstract

The current study aimed to evaluate the harmful effects of single and mixed minerals involved in water hardness on Nile tilapia (*Oreochromis niloticus*). Seventy-five fish were randomly assigned to five groups (*n* = 15), each consisting of three replicates (*n* = 5), and subjected to the following treatments for two months: standard fishery water, Ca (186 mg/L CaCl₂), Mg (40 mg/L MgCl₂), Na (25 g/L NaHCO₃), a mixture of MgCl₂, CaCl₂, and NaHCO₃ at the same protocol dosing to adjust water hardness at 300 mg/L. The results reported that fish exposed to Ca, Na, and Mix salts showed a significant decline in growth performance, serum antioxidant levels, and protein profile along with a significant rise in the enzymatic activity of ALT, AST, urea, creatinine, and MDA levels (*P* ≤ *0.05*). Exposure of fish to either single or mixed ions caused histopathological changes in the gills, liver, kidney, spleen, and brain. However, the nature, distribution, and the degree of severity of histopathological lesions vary in the experimental groups. The group exposed to mixed salts had the highest scores in all the pathological parameters, whereas the Mg group recorded the lowest scores. Moreover, the Ca group exhibited the highest scores in the liver and kidney, while the Na group showed the highest scores in the gills, spleen, and kidney.

**Supplementary Information:**

The online version contains supplementary material available at 10.1007/s10695-025-01532-9.

## Introduction

Nile tilapia is an important aquaculture species worldwide, recognized for its rapid development, substantial yields, adaptation to many farming environments, nutritional benefits, disease resistance, contribution to agricultural sustainability, and economic advantages (Geletu and Zhao 2023). Environmental contamination significantly affects aquaculture by disturbing ecosystems, compromising fish health, and diminishing production. Heavy metals are the predominant contaminants that adversely affect the physiological functions of numerous fish species. Many recent studies have demonstrated that extended exposure to heavy metals, including cadmium, lead, and copper could alter growth performance, as well as hematological and biochemical parameters in various aquaculture species such as *Oreochromis niloticus, Wallagu attu, Mystus seenghala,* and *Cyprinus carpio* (Fazio et al. 2022; Habib et al. 2024; Amouri et al. 2025; Rind et al. 2025). Water hardness may mitigate the toxicity of heavy metals in some aquatic species, while extremely hard water may have a detrimental effect on fish physiological activity as well as the productivity and reproductivity of fish (Boyd and Tucker 2012; Limbaugh et al. 2021; Swain et al. 2020).

Water hardness refers to the quantity of multivalent cations, which are often calcium (Ca^2+^) and magnesium (Mg^2+^) due to their extreme prevalence in natural freshwater systems (Baldisserotto 2011). Permanent hardness is caused by sulphates and chlorides of calcium and magnesium, while temporary hardness is chemically linked to the carbonate and bicarbonate of calcium and magnesium salts, such as CaCO3 (Boyd 2015). The location and source of the water can significantly affect the water hardness, which ranges from 0 to 60 mg/L as soft, 61 to 120 mg/L as moderately hard, and more than 180 mg/L as very hard (United States Geological Survey 2016). Wen et al. (2024) demonstrated that the source of water significantly influences water hardness, particularly in coastal areas where factors such as evaporation and seawater intrusion play a major role. Furthermore, geographical location also affects hardness levels, with a noticeable decrease in hardness as groundwater depth increases indicating reduced influence from human activities.

For aquaculture, a certain amount of water hardness (25–100 mg/L Ca^2+^) is often advised because Ca^2+^ has significant advantages and functions (Romano et al. 2020). Mg^2+^ is a vital intracellular divalent cation that plays a critical role in numerous physiological functions. It forms an essential complex with ATP, serving as a cofactor in many key biological processes, including protein synthesis, cell division, and energy metabolism (Lall and Kaushik 2021). In addition, sodium (Na⁺) and chloride (Cl⁻) are the two primary ions responsible for maintaining the hyperosmotic state in freshwater animals (Griffith 2017). The optimum level of NaHCO_3_ in water is 50—150 mg/L (Boyd 2020).

Excessive calcium deposition in vital organs can be detrimental to fish health and, in severe cases, may lead to mortality (Baldisserotto et al. 2014). Additionally, Swain et al. (2020) demonstrated that the water hardness affects the fish's reproductive behavior and physiology. CaCl_2_ quickly dissociates into calcium and chloride ions in water due to its low molecular weight and high-water solubility, which the intestines can absorb effectively (Bailone et al. 2022). Mallick et al. (2014) found extensive damage to eggs, spawn, fry, and fingerlings of fish when the concentration of CaCl_2_ in the water was elevated. Exposure to MgCl_2_ may have a detrimental effect on fish physiology and behavior because it causes a stress response in fish by raising cortisol levels (Riepe 2022). Harper et al. (2014) demonstrated the toxicity of sodium bicarbonate (NaHCO3) to seven different fish species. According to another study, high levels of sodium bicarbonate can have a detrimental effect on Nile tilapia growth performance parameters (Song et al. 2021). Moreover, high sodium bicarbonate exposure can impair splenic immune function in Nile tilapia (Zhao et al. 2022). Furthermore, exposure to sodium bicarbonate significantly lowers antioxidant enzyme levels (Li et al. 2022).

Based on the comparatively limited number of experimental investigations on the impact of water hardness on fish, the main mineral ions involved are Ca^2+^, Na^+^, Mg^2+^, and Cl^−^. Since little is known about the detrimental effects of water hardness on Nile tilapia and the relative importance of each component, the purpose of our study is to investigate the chronic negative impact of either individual or a mixture of CaCl_2_, MgCl_2_, and NaHCO3 on Nile tilapia by measuring growth performance, organ function biomarkers, oxidative stress markers, and histopathology of many organs.

## Materials and methods

### Chemicals, reagents, and kits

Calcium chloride (CaCl2, 99.9%) was purchased from Techno Pharm Chem, Bahadurarh, Haryana, India, Cat. **#**10043–52-4, Magnesium chloride (MgCl2, 98%) was purchased from Alpha Chemika, India, Cat. **#**7791–18-6, Sodium bicarbonate (NaHCO3, 99%) was purchased from Power Chemical, Quesna, Monufia, Egypt, Cat. **#**144–55-8. Malondialdehyde (Cat. # MD2529), Catalase (Cat. # CA 25 17), Reduced glutathione (Cat. # GR2511), Total protein (Cat. **#**TP 20 20), Albumin (Cat. #AB 10 10), Aspartate aminotransferase (AST, Cat. # AS 1061), Alanine transaminase (ALT, Cat. # AS 1031), Urea (Cat. # UR 21 10), Creatinine (Cat. # CR 12 50), Calcium (Ca, Cat. **#** CA 12 10), Magnesium (Mg, Cat. # MG 16 10), Sodium (Na, Cat. # SO 19 10), and Chloride (Cl, Cat. # CL 12 11) were purchased from Biodiagnostic Com., Cairo, Egypt.

### Preparation of hard water nominal to 300 ppm CaCo3

Hard water was prepared according to the Environmental Protection Agency (2019) as follows; stock solution A was prepared by dissolving 19.84 g anhydrous MgCl2 and 46.24 g CaCl2 in 1L de-ionized water, while stock solution B was prepared by dissolving 35.02 g NaHCO3 in 1L de-ionized water. Both stock solutions were sterilized and stored in the refrigerator till use. To prepare 1L of 300 ppm hard water, add 6.0 mL of solution A and 8.0 mL of solution B to 1L of de-ionized water, then mix and adjust the pH to 7.0 ± 0.2 by adding 1 N NaOH or 1 N HCl if necessary.

### Fish culturing and experimental design

All fish procedures were carried out in accordance with the institutional animal (fisheries) care and use committee (IACUC) guidelines set out by Cairo University (IACUC protocol number: Vet Cu 131020241015).

Seventy-five monosex Nile tilapia (*Oreochromis niloticus*) with an average weight of 72.2 ± 20 g/fish, and an age around 2–3 months, were acquired from a local fish farm (Kafr El-Shaikh governorate, Egypt). Fish were housed in 90-L glass tanks with constant aeration and under laboratory conditions. To ensure their general health, the fish were acclimated for fourteen days. The fish were fed a commercial feed, which was composed of the following nutritional ingredients: 30% crude protein, 6.29% crude fibers, 6% lipid, 65% fish meal, 3990 kcal, 60% gluten, 60% choline chloride, wheat bran, yellow corn, 46% soybean meal, mono calcium phosphate, distillers dried grains with soluble (DDGS), vitamin and mineral premix (Skretting Com., El Sharqia, Egypt). This commercial food was manually fed to fish twice a day (7:30 and 17:30) at a rate of 2% of their body mass until they were satiated. The experiment was conducted for two months.

Fish were divided randomly into five experimental groups after the acclimatization period (*n* = 15 fish per group). Each group was divided into three replicates (3 tanks were used for each group with 5 fish per tank). Group (1) served as the control group (the baseline hardness is below 180 mg CaCO_3_ L^−1^), group (2) was the Ca group (water hardness was adjusted to 200–250 mg/L by adding 186 mg/L stock solution of CaCl_2_), group (3) was the Mg group (water hardness was adjusted to 150–180 mg/L by adding 40 mg/L stock solution of MgCl_2_), group (4) was the Na group (water hardness was adjusted to 120–150 mg/L by adding 25 g/L of NaHCO_3_), and group (5) was the Mix salts group (water hardness was adjusted to 300 mg/L by adding stock solution of three chemicals MgCl_2_, CaCl_2_, and NaHCO_3_ according to the Environmental Protection Agency (2019) and were monitored (correction done if needed). Different water hardness levels were attained by mixing chemicals with fishery water. Water chemistry testing was done before and after exposure to mineral ions. Following Zhang et al. (2022), the water in the tanks was replaced daily during the experiment to maintain a constant hardness concentration. The alkalinity concentration was also monitored continuously at experimental levels throughout the exposure period. All fish were checked every day for any unusual clinical symptoms or deaths.

### Water quality measurements

During the experiment, water quality parameters were maintained within a suitable range for Tilapia. Water temperature and dissolved oxygen averaged 28.00 ± 1.00 ◦ C and > 5 mg/L, respectively; ammonium, nitrite, and nitrate averaged 0.06 mg/L, 0.05 mg/L, and 55 mg/L, respectively. All these water quality parameters were within acceptable ranges for fish growth (Boyd 1979). During the experimental study, water samples were collected weekly from each aquarium. The water physical–chemical variables were monitored weekly for pH, EC, and TDS using a digital Mini-pH Meter, model 55, USA. Hardness, Alkalinity, and chloride by a titration method according to Eaton et al. (2005) and un-ionized ammonia (Colt 2002).

### Growth performance

To determine the initial, weekly, and final body weights (IBW, GW, and FBW), each fish in each group was weighed separately. During the experiment, the following calculations were made for weight gain (WG), feed conversion ratio (FCR), and specific growth rate (SGR %) evaluations (Abdel Rahman et al. 2021) as follows:

$$\text{SGR}\left(\%\right)\left\{\text{LN}\left(\text{FBW}\right)-\text{LN}\left(\text{IBW}\right)/\text{time}\right\}*100,$$

where

LN: natural log

$$\begin{array}{c}\text{WG }\left(\text{g}/\text{fish}\right)=\text{FBW}-\text{IBW}.\\ \text{FCR}=\text{Dry feed fed }\left(\text{g}\right)/\text{WG}\left(\text{g}\right).\end{array}$$

### Sampling

At the end of the experiment, blood samples were collected from the tail vein of all fish in each group. To obtain clear serum samples, centrifugation was performed for five minutes at 4,500 RPM. The samples were then stored at −20°C until they were required for biochemical testing. The fish were then anesthetized with 20 mg/L of clove oil (Akinjogunla et al. 2023), and their internal organs were checked for abnormalities. After that, samples from different internal organs (gills, liver, kidney, spleen, and brain) were collected and preserved for histopathology in 10% neutral buffered formalin.

### Ions content, organ function test, and oxidative stress evaluation

Calcium (Ca^2+^), magnesium (Mg^2+^), sodium (Na^+^), chloride (Cl^−^), ALT, AST, total protein, albumin, urea, and creatinine, malondialdehyde (MDA), catalase (CAT), and reduced glutathione (GSH) were determined using serum samples in accordance with the guidelines provided by commercial kits (Biodiagnostic Co., Giza, Egypt).

### Pathological examination

Formalin-fixed tissue samples were dehydrated through a series of graded ethyl alcohol solutions (50–100%), cleared with two changes of xylene, and embedded in molten paraffin wax. The samples were then stained with H&E and examined under an Olympus BX43 light microscope to detect any abnormalities. Using an Olympus BX43 light microscope, the formalin-fixed tissue samples were dehydrated using graded ethyl alcohol (50–100%), cleaned with two xylene changes, embedded in molten paraffin wax, and stained with H&E to look for any anomalies. Certain sections were stained with both Congo red to identify eosinophilic inflammatory cells, and Masson's trichrome (MTC) to highlight collagen fibers. All slides were photographed for histopathology grading using an Olympus DP27 camera with Olympus CellSens dimensions software (Bancroft and Gamble 2013).

In accordance with the methodology described by Bernet et al. (1999), the degree of histological abnormalities in each examined organ was assessed using a quantitative parametric grading system. This system relies on how pathogenic alterations develop and how significant they are. Depending on the severity of the lesion, each modification is assigned a number between 0 and 6: (0) unaffected, (1–2) mild incidence, (3–4) moderate manifestation, and (5–6) severe manifestation (diffuse lesion). However, the following three significant parameters are applied to classify the changes based on their pathological relevance: (1) low, where the injury can be easily repaired if the cause is eliminated; (2) moderate, where the lesion is usually reversible if the stressor is neutralized; and (3) severe, where the lesion is significantly pathologically significant, usually permanent, and causes partial or total loss of organ function. The sum of the products of two components of each alteration is utilized to evaluate the histological lesions in each organ using statistical analysis.

### Statistical analysis

The SPSS program was used to analyze all the data, which were presented as means ± standard error of the mean (SEM) and graphs were drafted using GraphPad Prism software (version 8. 4. 3 (686), California, 2020). One-way analysis of variance (ANOVA) and Tukey's test were used to compare results from various groups. *P values* ≤ *0.05* indicate statistical significance.

## Results

### Water quality measurements

**Fig. 1:**
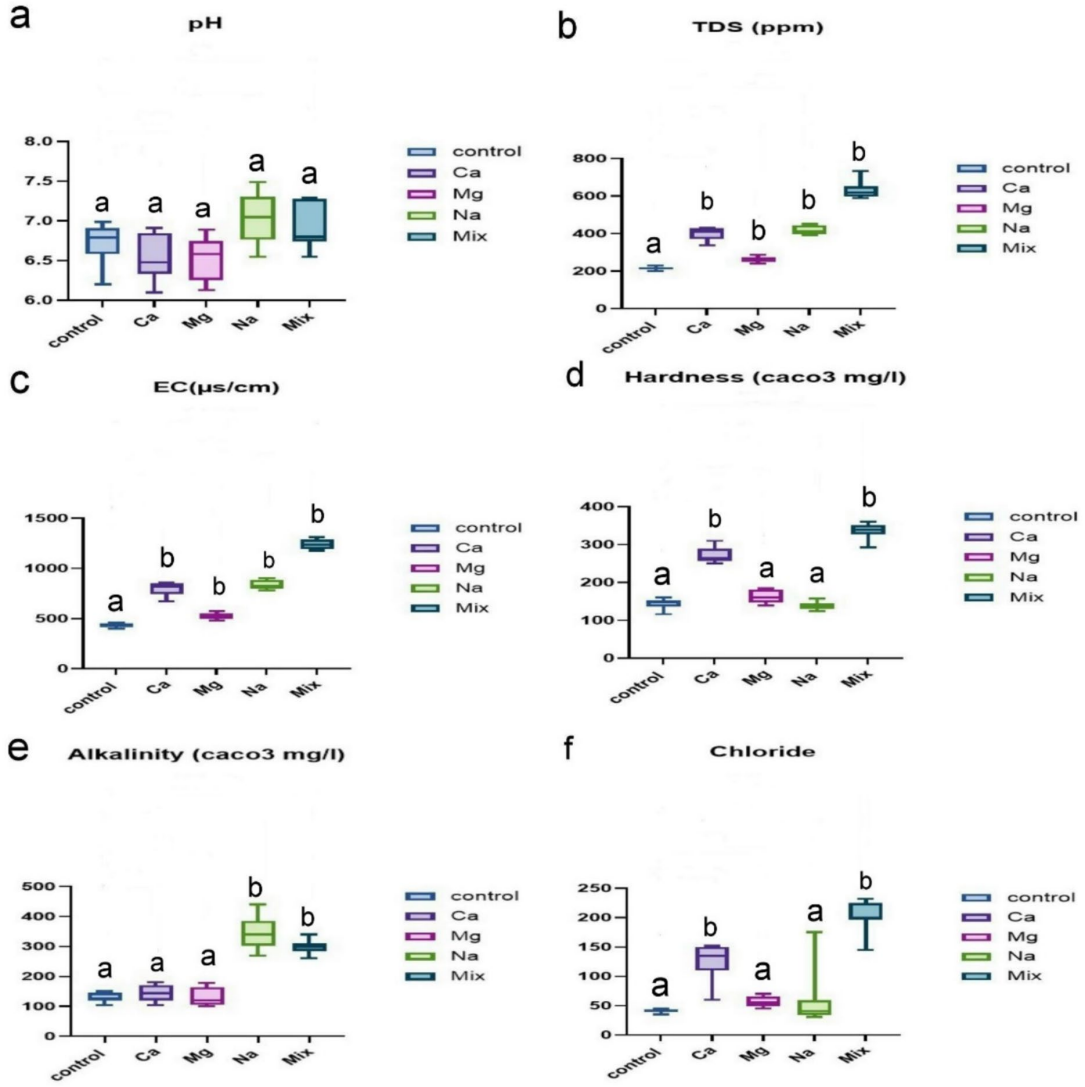
Bar graph shows the various water quality parameters. **a** PH, **b** EC, **c** TDS, **d** hardness, **e** alkalinity, **f** chloride. Note that, EC; electrical conductivity, TDS; total dissolved solids. * indicates statistical significance at *P* ≤ *0.05*, whereas ns indicates non-significant results

The following water physical–chemical variables were monitored daily, dissolved oxygen > 5 mg/L and temperature (27–29 °C) were kept nearly constant by artificial control. Ammonia < 0.05 mg/L during the experimental period. The pH did not differ significantly between groups. However, all hardness-exposed groups showed a significant rise in some water parameters, including EC and TDS, when compared to the control group. Furthermore, the hardness and chloride levels significantly increased in the Ca and Mix exposed groups, while the Mg and Na receiving groups showed no significant difference from the control group. Furthermore, water alkalinity significantly increased in the Na group compared to the control group (Fig. 1).

### Growth performance

Table 1 showed a significant rise in FCR and a decrease in FBW, SGR, and WG in the Ca, Na, and Mix salts groups compared to the control group. However, there was no significant difference between the control group and the Mg group in all examined growth performance parameters.

**Table 1:** Assessment of growth performance parameters in *O. niloticus* following different treatments

	Control	Ca	Mg	Na	Mix salts
IBW (g)	71.40 ± 0.73	73.80 ± 0.30	76.40 ± 1.94	74.60 ± 1.25	78.60 ± 1.13
FBW (g)	149.40 ± 5.76^a^	91.20 ± 1.10^b^	119.60 ± 0.34^a^	105.00 ± 0.63^b^	100.40 ± 3.91^b^
WG (g)	78.00 ± 0.37^a^	17.40 ± 1.50^b^	44.80 ± 0.33^a^	30.40 ± 5.42^b^	21.80 ± 3.42^b^
SGR (%)	1.06 ± 0.17^a^	0.44 ± 0.10^b^	0.64 ± 0.14^a^	0.29 ± 0.04^b^	0.52 ± 0.08^b^
FCR	0.80 ± 0.04^a^	4.47 ± 0.20^b^	1.65 ± 0.10^a^	2.63 ± 0.30^b^	3.35 ± 0.19^b^

Values were shown as mean ± SEM (*n* = 15). In the same row, small litters mean a significant difference at *P* ≤ *0.05*. Abbreviations: *IBW* initial body weight, *FBW* final body weight, *WG* weight gain, *SGR* specific growth rate, *FCR* feed conversion ratio

### Ions content, organ function test, and oxidative stress evaluation

Compared to the control group, the groups exposed to Ca, Mg, and Na treatments showed significantly higher serum levels of Ca^2^⁺, Mg^2^⁺, and Na⁺ ions, respectively. All hardness-exposed groups exhibited elevated serum Cl⁻ levels except the Na group. Additionally, the group exposed to the Mix salts displayed significantly higher levels of Ca^2^⁺, Mg^2^⁺, Na⁺, and Cl^−^ ions when compared to the control group (Table 2).

**Table 2:** Changes in serum ion content of *O. niloticus* under different water hardness treatments

	Control	Ca	Mg	Na	Mix salts
Ca^2^ + (mmol/L)	3.11 ± 0.60^a^	6.60 ± 1.40^b^	3.13 ± 0.04^a^	2.99 ± 0.04^a^	6.25 ± 0.66^b^
Mg^2^ + (mmol/L)	1.54 ± 0.01^a^	1.25 ± 0.01^a^	5.99 ± 1.24^b^	1.40 ± 0.09^a^	4.61 ± 0.38^b^
Na^+^ (mmol/L)	281.30 ± 0.34^a^	220.43 ± 0.16^a^	157.00 ± 0.51^a^	526.06 ± 0.62^b^	513.86 ± 0.18^b^
Cl^−^ (mmol/L)	131.20 ± 2.27^a^	161.93 ± 0.43^b^	157.60 ± 0.20^b^	144.63 ± 1.70^a^	174.26 ± 0.33^c^

Values were shown as mean ± SEM (*n* = 15). In the same row, small litters mean a significant difference at *P* ≤ *0.05*

Serum activity/level of ALT, AST, urea, and creatinine were significantly higher in all hardness-exposed groups than in the control group, but total protein and albumin levels were lower (Table 3).

**Table 3:** Response of organ biomarker levels in *O. niloticus* to different water hardness treatments

	Control	Ca	Mg	Na	Mix salts
ALT (U/mL)	33.86 ± 0.69^a^	91.12 ± 0.59^b^	71.98 ± 0.63^b^	88.01 ± 0.62^b^	94.65 ± 0.04^b^
AST (U/mL)	40.99 ± 2.32^a^	89.15 ± 0.28^b^	79.12 ± 1.19^c^	86.17 ± 0.34^b^	92.72 ± 2.64^c^
Alb (g/dL)	1.34 ± 0.07^a^	0.66 ± 0.06^b^	0.76 ± 0.01^c^	0.70 ± 0.05^b^	0.51 ± 0.19^b^
TP (g/dL)	3.03 ± 0.20^a^	1.76 ± 0.37^b^	1.86 ± 0.20^b^	1.70 ± 0.17^b^	1.39 ± 0.13^c^
BUN (mg/dL)	4.05 ± 0.65^a^	8.24 ± 0.89^b^	7.30 ± 0.57^b^	7.31 ± 0.39^b^	8.55 ± 0.81^c^
Creatinine (mg/dL)	0.43 ± 0.12^a^	1.49 ± 0.22^b^	1.46 ± 0.30^b^	1.59 ± 0.31^b^	1.94 ± 0.01^c^

Values were shown as mean ± SEM (*n* = 15). In the same row, small litters mean a significant difference at *P* ≤ *0.05*

According to the results in the Table 4, the group that received Ca, Mg, Na, and mix had significantly greater MDA level and lower GSH and CAT activity than the control group.

**Table 4:** Alterations in oxidative stress biomarkers of *O. niloticus* under varying water hardness treatments

	MDA (mmoL/mL)	CAT (U/L)	GSH (mg/dL)
Control	8.23 ± 0.23^a^	200.96 ± 0.26^a^	1.56 ± 0.07^a^
Ca	25.43 ± 2.87^b^	93.52 ± 0.66^b^	1.08 ± 0.04^b^
Mg	18.90 ± 1.51^b^	100.47 ± 0.15^b^	1.16 ± 0.08^c^
Na	20.13 ± 2.82^b^	99.62 ± 0.29^b^	1.10 ± 0.25^b^
Mix salts	26.96 ± 1.77^b^	91.69 ± 0.60^b^	1.07 ± 0.10^b^

Values were shown as mean ± SEM (*n* = 15). In the same column, small litters mean significant difference at *P* ≤ *0.05*

### Pathological examination

#### Clinical signs, gross findings, and mortalities

There were not any obvious abnormalities in both the control and Mg groups. On the other hand, the Ca-treated fish displayed black spots, ulcers, loss of scales, and a shredded tail and pectoral fins. Furthermore, one fish died among 15 (mortality rate ≈ 7%). The Na-treated fish showed darkness, aggregation around the air source, exophthalmia, hemorrhagic streaks and erosion in the pectoral fins, and red nodules/vesicles on the lower lip. While Mix-exposed fish demonstrated sever darkness, abnormal swimming patterns, exophthalmia, abnormal balance, hemorrhagic streaks, and nodules on the pectoral fins. The internal organs of all hardness-exposed groups showed atrophied spleen along with the presence of white spots on the liver in some fish (indicating a mottled liver), while others exhibited black patches on the liver (Fig. S1).

#### Histopathological finding

**Fig. 2:**
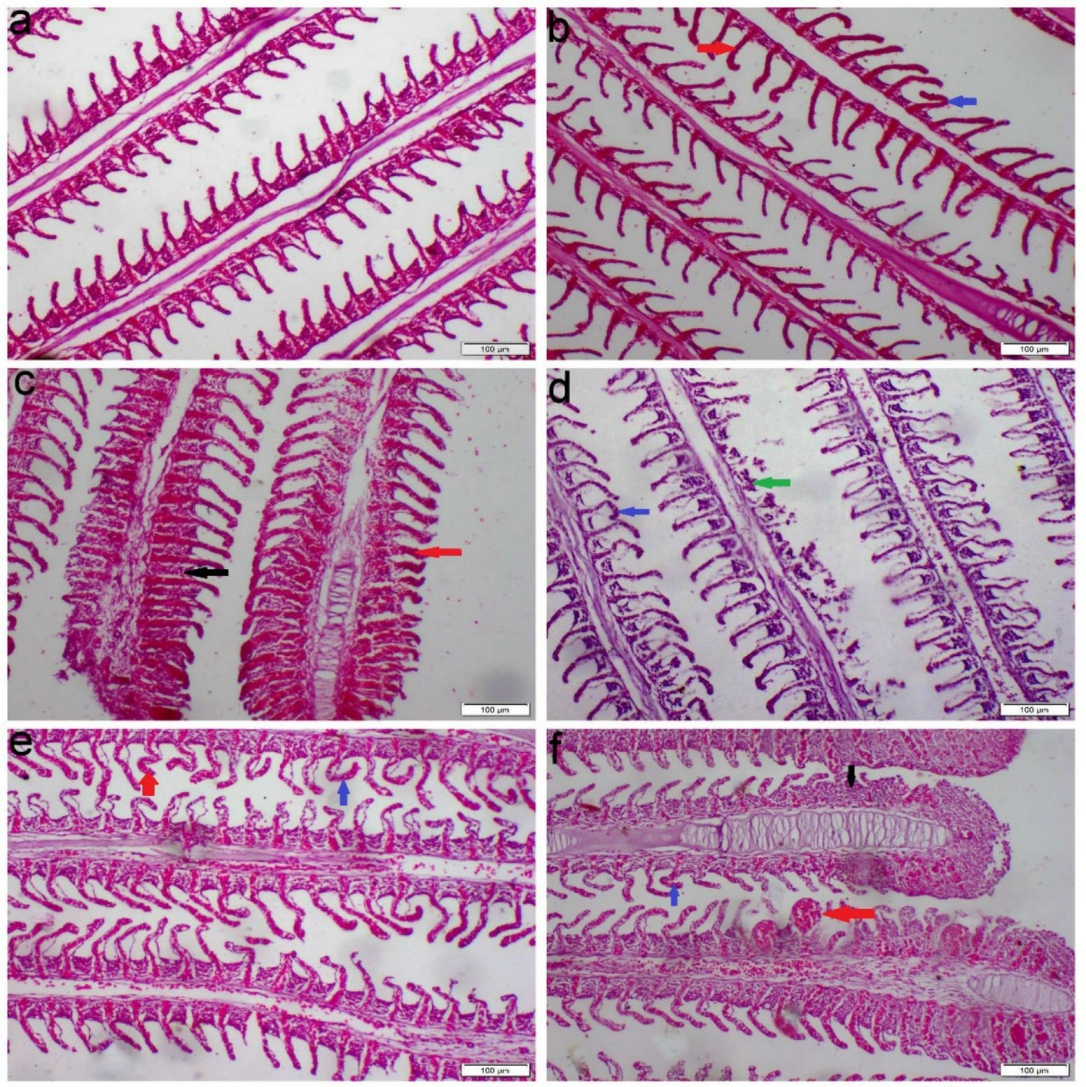
Photomicrograph of gill tissue sections stained with H&E. **a** Control group, **b** Ca group, **c** Mg group, **d** Na group, **e**–**f** Mix salts group. (red arrow) congestion and telangiectasis; (black arrow) lamellar hyperplasia; (blue arrow) curling of gill lamellae; (green arrow) necrosis of gill lamellae

**Fig. 3:**
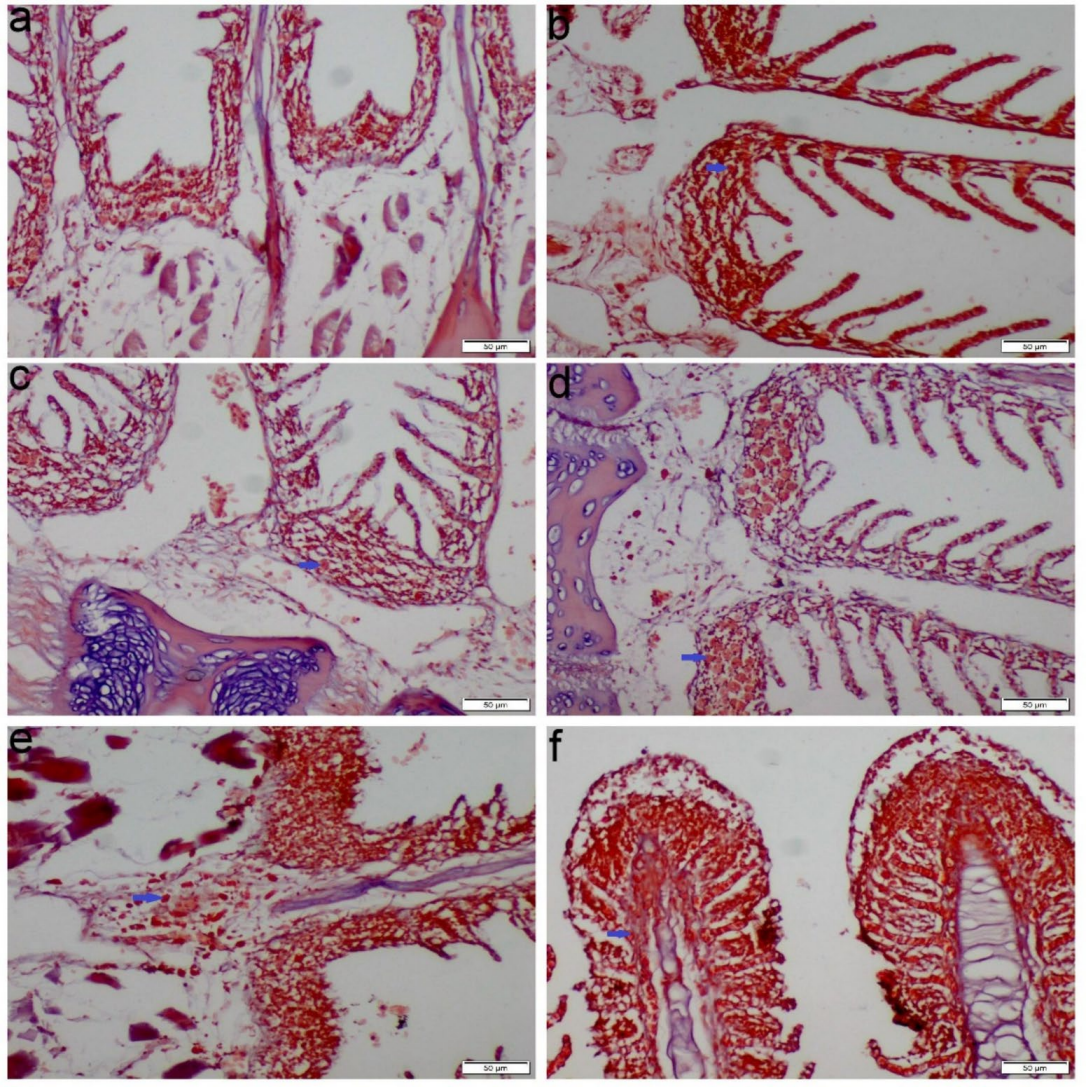
Photomicrograph of gills tissue sections stained with Congo red. **a** Control group, **b** Ca group, **c** Mg group, **d** Na group, **e**–**f** Mix salts group. (blue arrow) orange red-stained eosinophils cells

The gill tissues of the control group displayed a normal histological structure, with well-preserved primary and secondary lamellae. In contrast, fish exposed to either single or mixed minerals exhibited vascular congestion and curling of the epithelial lining in both primary and secondary lamellae. Additionally, exposure to Mg resulted in epithelial hyperplasia and lamellar fusion. Moreover, the Na group showed significant necrosis in the secondary lamellae (Fig. 2). Congo red staining revealed a distinct accumulation of eosinophilic inflammatory cells in all groups exposed to water hardness, predominantly concentrated at the base of the primary lamellae and gill arch (Fig. 3).

**Fig. 4:**
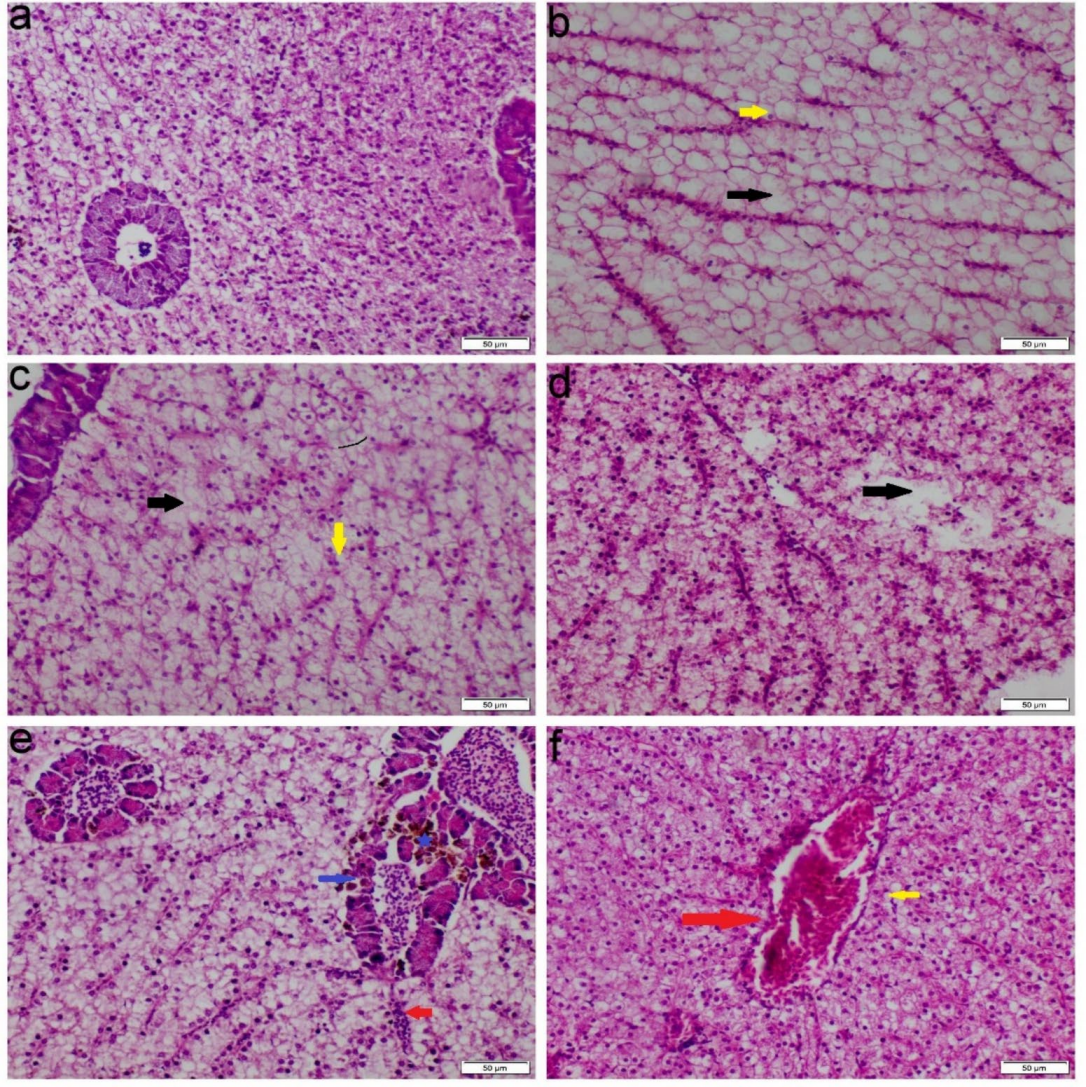
Photomicrograph of liver tissue sections stained with H&E. **a** Control group, **b** Ca group, **c** Mg group, **d**-**e** Na group, **f** Mix salts group. (red arrow) congestion of blood vessels; (black arrow) necrosis of hepatocytes; (blue arrow) necrosis of pancreatic cells; (green stars) accumulation of melanophores; (yellow arrow) vacuolar degeneration of hepatocytes

The liver of the control group showed normal histological features, characterized by intact hepatocytes and well-defined sinusoidal structures. In contrast, fish exposed to either single or mixed ions showed various degrees of hepatopancreatic degeneration and necrosis. There were vascular congestion and brown pigments aggregation throughout the hepatopancreatic tissue and within macrophages. These lesions were more pronounced in the Ca, Na, and Mix groups, but less severe in the Mg group. Additionally, both Ca and Mix groups demonstrated notable infiltration of mononuclear cells throughout the hepatic parenchyma (Fig. 4).

**Fig. 5:**
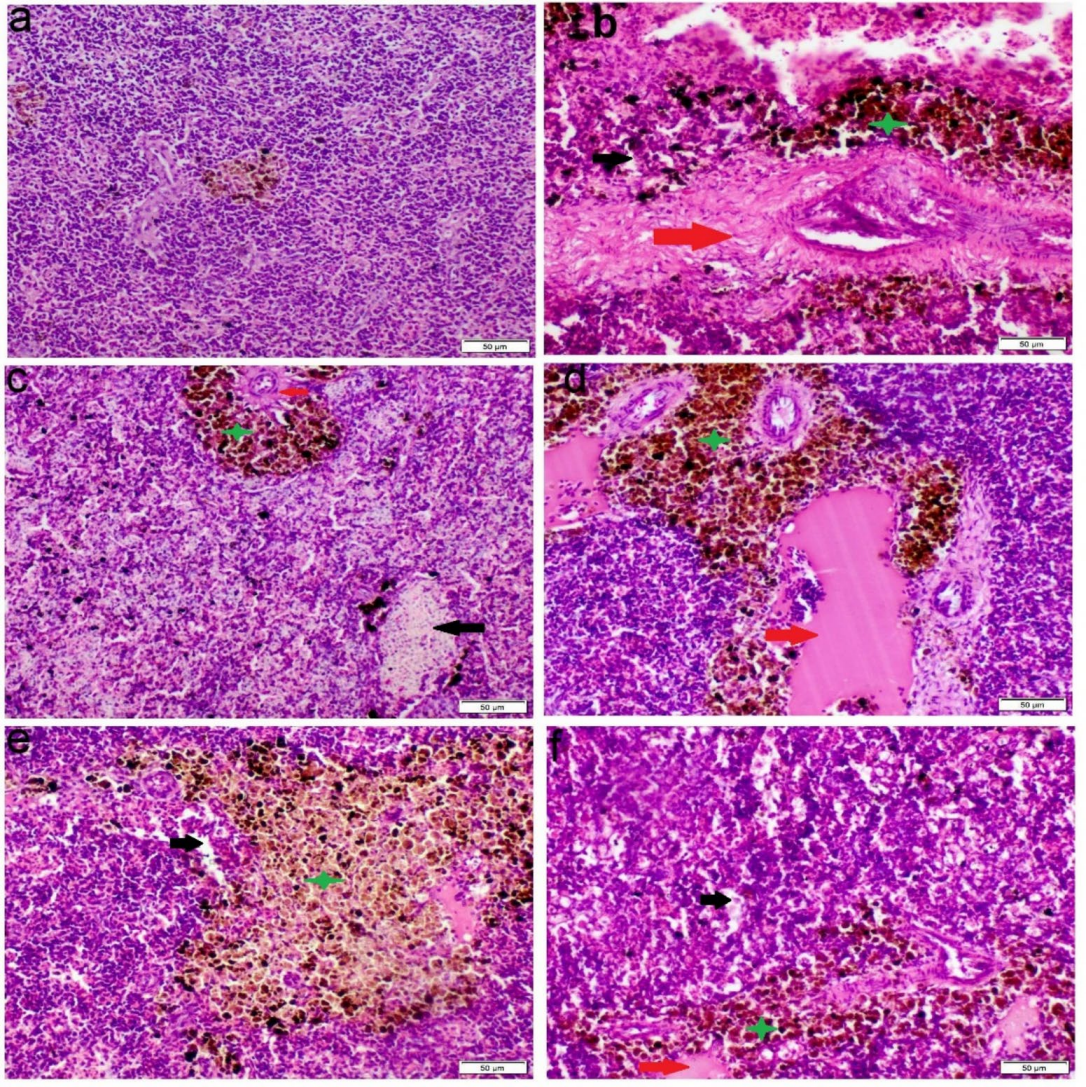
Photomicrograph of spleen tissue sections stained with H&E. **a** Control group, **b** Ca group, **c** Mg group, **d**-**e** Na group, **f** Mix salts group. (black arrow) depletion of lymphocytic tissue; (green stars) melanomacrophage center activation; (red arrow) perivascular edema

The spleens of the control group exhibited normal histological architecture. In contrast, spleens obtained from both Ca and Mg exposed groups showed pronounced lymphocytic depletion, perivascular edema, and activation of the melanomacrophage centers. While Mg exposed group showed mild lymphoid depletion in some sections. The group exposed to Mix salts exhibited all of these pathological changes with greater severity (Fig. 5).

**Fig. 6:**
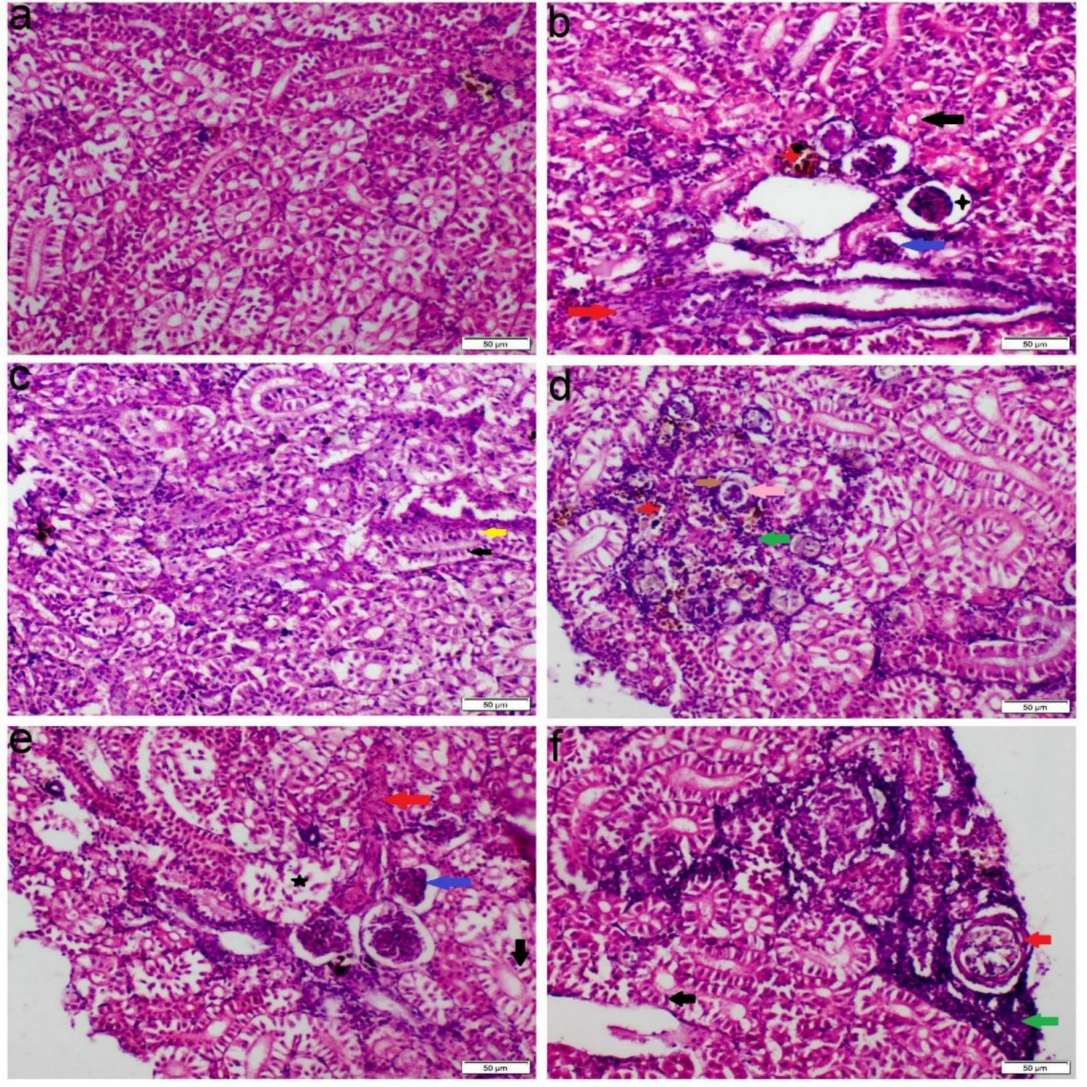
Photomicrograph of kidney tissue sections stained with H&E. **a** Control group, **b** Ca group, **c** Mg group, **d**-**e** Na group, **f** Mix salts group. (black arrow) coagulative necrosis of renal tubular epithelium; (yellow arrow) vacuolar degeneration of renal tubular epithelium; (green arrow) leucocytes inflammatory cells infiltration; (blue arrow) atrophy of glomerular tuft; (pink arrow) structural abnormality; (red stars) melanomacrophage center activation; (black star) widening of bowman’s capsules; (brown arrow) thickening of bowman’s capsule; (red arrow) fibrosis

**Fig. 7:**
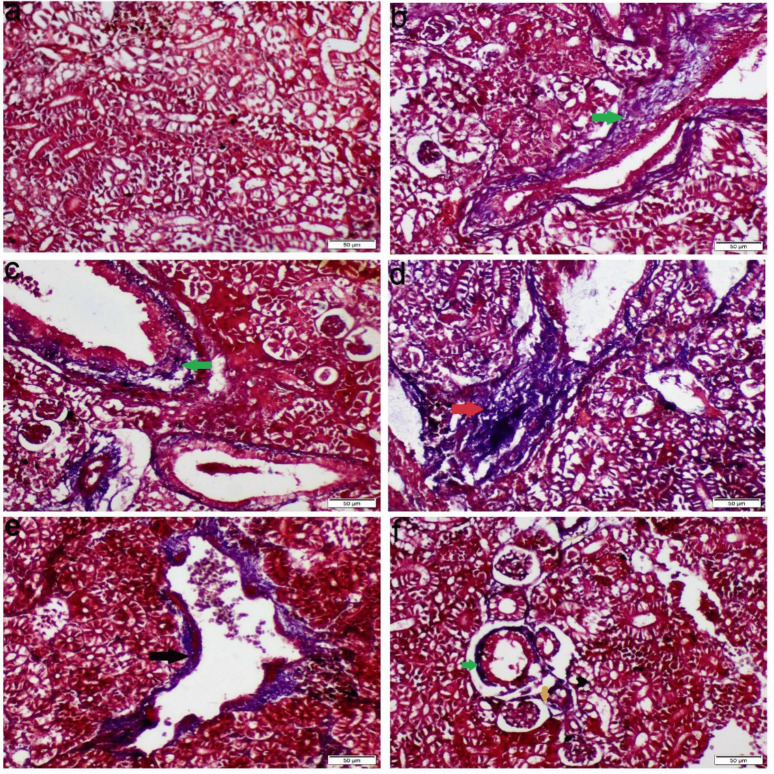
Photomicrograph of kidney tissue sections stained with MTC. **a** Control group, **b** Ca group, **c** Mg group, **d** Na group, **e**–**f** Mix salts group. (green arrow) blue-stained collagen fibers around renal tubules; (yellow arrow) blue-stained collagen fibers around bowman’s capsule; (red arrow) blue-stained collagen fibers in the interstitial space; (black arrow) blue-stained collagen fibers around blood vessels

 Compared to the control group, kidney tissues from the Ca-exposed fish exhibited mononuclear cell infiltration, vacuolar degeneration, prominent coagulative necrosis of the tubular epithelial cells, and activation of melanomacrophage centers. Furthermore, the Mg-exposed group showed mild vacuolar degeneration and necrosis of some renal tubular epithelium. Moreover, the Na group showed glomerular damage, structural abnormalities, and atrophy of the glomerular tuft with a widening of the Bowman’s space and thickening of the bowman’s capsule. Some glomeruli displayed complete necrosis and were replaced by chronic inflammatory cells (Fig. 6). The Mix salts group exhibited all the aforementioned pathological alterations with a more severe extent than those receiving the single ions (Fig. 6). MTC staining demonstrated blue-stained mature collagen fibers in the interstitial tissue, vascular wall, and bowman’s capsules (Fig. 7).

**Fig. 8:**
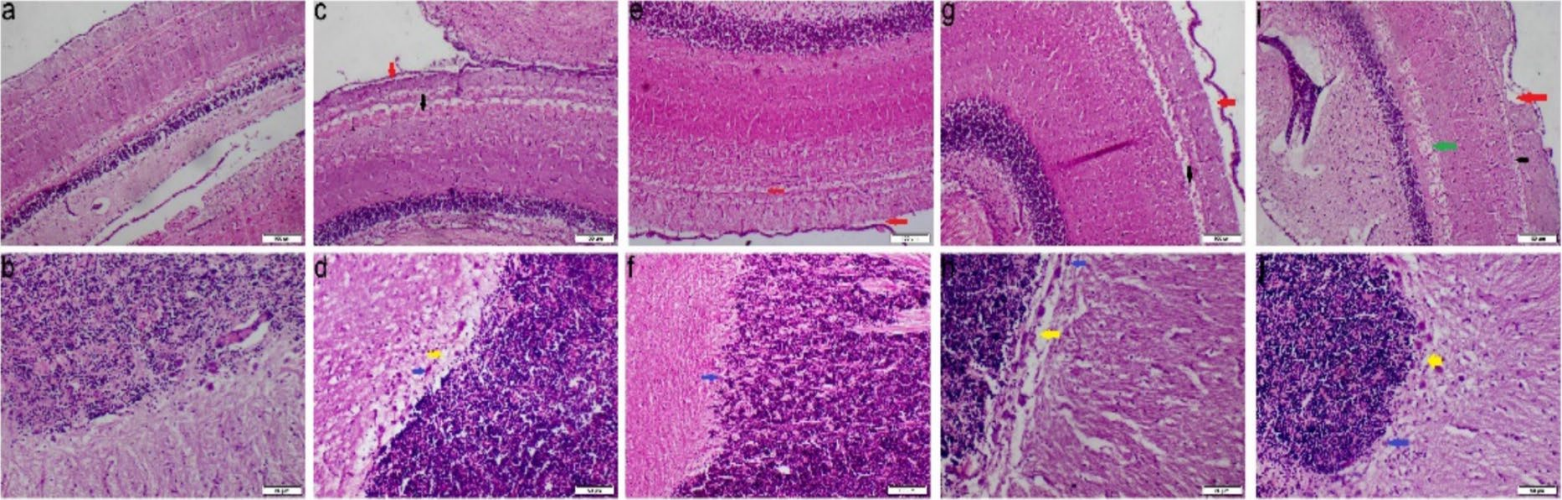
Photomicrograph of brain tissue sections at mesencephalon (optic tectum) and telencephalon (cerebellum) stained with H&E. **a**-**b** Control group, **c**-**d** Ca group, **e**–**f** Mg group, **g**-**h** Na group, **i**-**j** Mix salts group. (red arrow) dissociation of optic tectum layers; (black arrow) degeneration of optic tectum; (yellow arrow) vacuolation of Purkinje cell layers; (blue arrow) necrosis of Purkinje cells

The brain sections of the control group exhibited a normal histological structure in the optic tectum (OT) and cerebellum. In contrast, all experimental groups showed various degrees of pathological lesions in the examined areas. The OT displayed neuronal degeneration, vacuolation, and dissociation in both the upper and lower layers. Additionally, the cerebellum exhibited degeneration and necrosis of Purkinje cells along with vacuolation (Fig. 8).

**Fig. 9:**
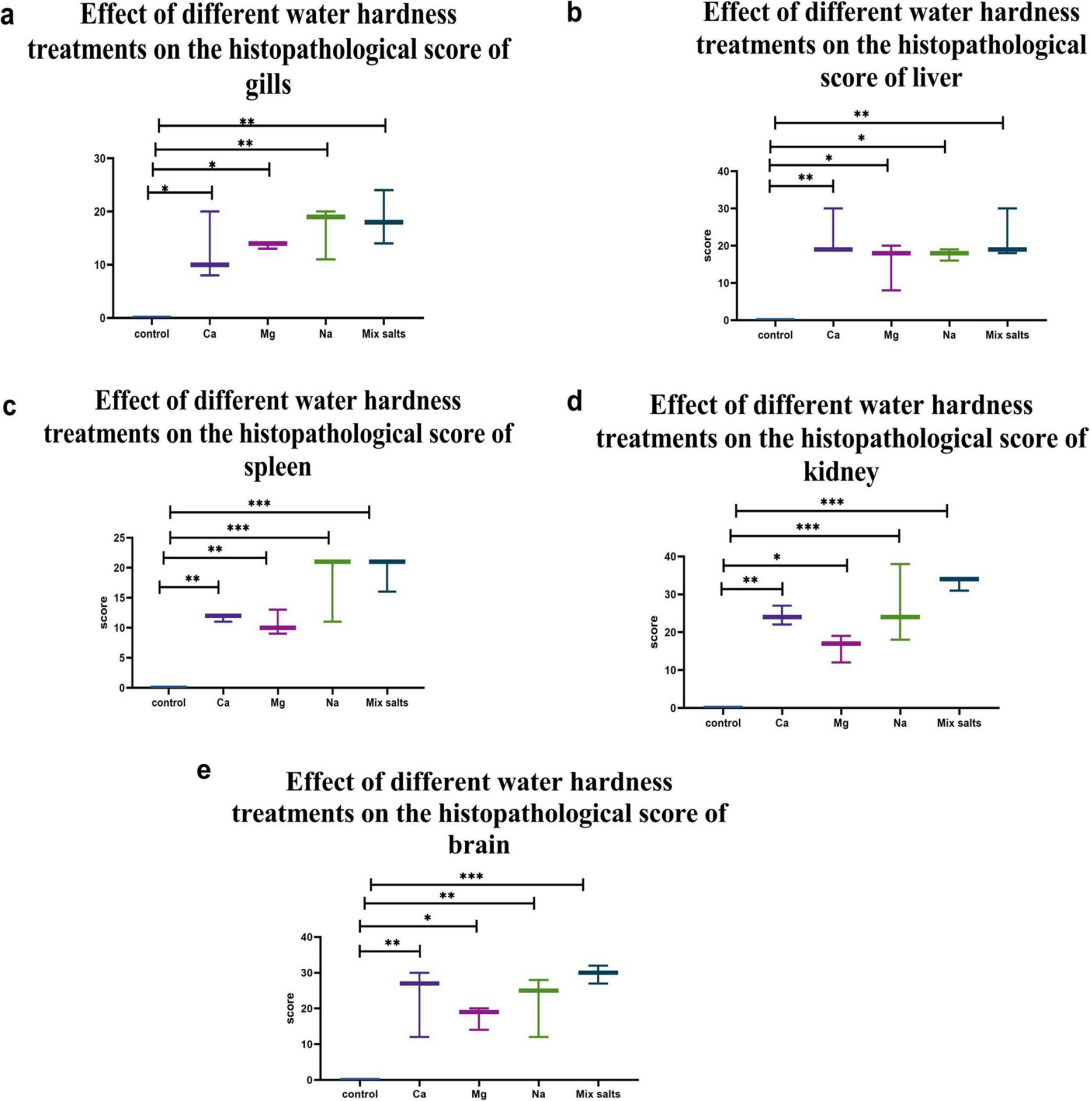
Bar chart demonstrating the effect of different water hardness treatments on histopathological lesion score index of different organs in *O. niloticus.* **a** gills, **b** liver, **c** spleen, **d** kidney, **e** brain. Note, Values were presented as mean ± SEM (*n* = 15/group). (*) statistically significant at *P* ≤ *0.05*, (**) statistically significant at *P* ≤ *0.01*, (***) statistically significant at *P* ≤ *0.001*

The microscopic lesion scores for various organs are presented in Fig. 9. The groups exposed to Mix salts had the highest scores in all assessed parameters, whereas the Mg group recorded the lowest scores. Moreover, the Ca-exposed group exhibited the highest histopathological scores in the liver and kidney, whereas the Na-exposed group showed the highest scores in the gills, spleen, and kidney.

## Discussion

The water hardness differs widely based on its source and is mainly influenced by the overall concentration of divalent cations, with Ca^2^⁺ and Mg^2^⁺ being the most dominant (de Holanda Cavalcante et al. 2014). The current study gives a comprehensive insight into the hazardous effects of chronic exposure of Nile tilapia to 300 mg/L hard water on growth performance, redox status, organ function parameters, and the microscopic appearance of most organs. Throughout the experimental period, all physicochemical water parameters such as dissolved oxygen, temperature, and ammonia levels were monitored daily. These parameters were within the acceptable ranges for Nile tilapia growth (Boyd 2017). All hardness-exposed groups exhibited elevated levels of EC and TDS compared to the control group because of increasing levels of dissolved ions. EC measures a liquid^’^s ability to conduct an electric charge and depends on the concentrations of dissolved ions which are expressed as TDS (Rusydi 2018). Furthermore, both hardness and chloride levels were significantly higher in the Ca and Mix groups than in others. This increase is attributed to the action of CaCl₂, which is known to elevate water hardness levels and chloride levels (Elphick et al. 2011). In contrast, water alkalinity was significantly elevated in both Na and Mix groups which is consistent with the observations of Furtado et al. (2011), who reported that NaHCO_3_ is an effective supplement for enhancing alkalinity.

The Ca, Na, and Mix exposed groups in our study showed a greater FCR and lower FBW, SGR, and WG values while the Mg-exposed group did not significantly vary from the control group. These results agreed with Michelotti et al. (2018) that demonstrated the toxic effect of CaCl_2_ on growth performance. Additionally, Song et al. (2021) demonstrated the negative impact of NaHCO_3_ on the growth performance of Nile tilapia. Our findings also showed abnormal behavioral signs in all hardness-exposed groups, except the Mg-receiving group. Mg^2+^ act as a calcium channel blocker, preventing the entrance of Ca^2+^ ions to the cell that makes fish to be calm (Sangiacomo et al. 2023). Furthermore, some fish from both the Na and Mix groups exhibited vesicles on the lower lip and pectoral fins. We propose that these vesicles result from irritation caused by NaHCO_3_ exposure. This aligns with the findings of Akinjogunla et al. (2023) who revealed that NaHCO_3_ causes irritation in fish.

The results of the present study showed elevated serum levels of Ca^2^⁺, Mg^2^⁺, Na⁺ in Ca, Mg, and Na-exposed group, respectively. While serum Cl⁻ levels were significantly elevated in Ca, Mg, and Mix salts exposed groups. This can be explained by the dissociation of CaCl₂ and MgCl₂ into Ca^2^⁺, Mg^2^⁺, and Cl⁻ ions, respectively, upon dissolution in water (Friesen et al. 2019). Similarly, NaHCO₃ dissociates into Na⁺ and HCO₃⁻ ions (Kontsevoi 2023). In aquatic organisms, gills are specialized for the active transport of various ions, including Ca^2^⁺, Mg^2^⁺, Na⁺, and Cl⁻, which play critical roles in acid–base balance and osmoregulation (Evans et al. 2005). These physiological mechanisms align with our findings of elevated serum ion concentrations. Changes in water hardness, specifically the concentration of Ca^2^⁺, can influence both Ca^2^⁺ and Na⁺ transport in the gills, as well as the associated uptake mechanisms. These alterations in ion transport are crucial because they can disrupt ion homeostasis and the balance of essential ions in the fish body (Limbaugh et al. 2021).

Our results indicated that all hardness exposed groups altered both liver and kidney function biomarkers. This was in line with several recent studies which showed that the toxicity of CaCl_2_, MgCl_2_, and NaHCO_3_ damages the liver and releases its enzymes into the blood (Ahmad et al. 2023; Chandra and Sukumaran 2020; Lei et al. 2024). The liver is the main organ of detoxification, and any rise in its enzymes is a sign of liver damage (Mahboub and Shaheen 2021). A rise in serum creatinine or BUN is indicative of kidney disease, and both creatinine and urea are valid markers of renal function (Ajeniyi and Solomon 2014; Ding et al. 2023). Our study also revealed that the long-term exposure of Nile tilapia to either single or a mixture of ions markedly altered oxidant/antioxidant balance manifested by increasing MDA level and reducing GSH and CAT activity. Under normal physiological conditions, free radicals are produced and removed to maintain a dynamic equilibrium (Ming et al. 2012). Antioxidant enzymes like CAT and GSH serve as cells'first line of defense against hazardous free radicals that induce oxidative stress (Hegazi et al. 2010). Our findings concurred with those of Vargas-Ramella et al. (2025) and Liu et al. (2025) who demonstrated that the CaCl_2_, MgCl_2_, and NaHCO_3_ treatment accelerated lipid oxidation and suppressed antioxidant enzymes.

Histopathological changes are thought to be useful markers for evaluating any negative impact of waterborne abnormalities, providing information about tissue damage, remodeling, and overall health. Nevertheless, little is known about the histopathological alterations in fish response to hard water. Our histopathological analysis of gill sections obtained from Ca, Na, and Mix group revealed severe congestion, necrosis of gill lamellae and hyperplasia. The significant necrosis of the lamellae, particularly in the Na-treated group, led to a notable aggregation of fish around the air source. This finding is consistent with the study by Farag and Harper (2014), which demonstrated that NaHCO₃ causes severe necrosis in fish gills. Eosinophils can be distinctly stained an orange-red color using Congo red, making them easily distinguishable from surrounding cellular structures (Song et al. 2018). The accumulation of eosinophils in gill tissues is commonly used as a histopathological marker to assess the severity of environmental pollutants and other stressors (Strzyzewska et al. 2016). Additionally, the liver exhibited severe vacuolar degeneration, necrosis, and chronic inflammatory responses. These findings are consistent with those of Wen et al. (2023) and Limbaugh et al. (2021), who also reported the hepatotoxic effects of NaHCO₃ and CaCl₂. Furthermore, our results showed that the kidney displayed necrosis, glomerular atrophy, and chronic inflammation, aligning with the findings of Ding et al. (2023), which highlighted the harmful impact of NaHCO₃ on renal tissue.

MTC is a special staining technique that provides contrasting colors to collagen (blue) and muscle fibers (brown), allowing for clearer visual differentiation between the two tissue components (Mishra et al. 2015). These findings were consistent with the oxidative and biochemical results. In contrast, the Mg group showed a lower degree of histopathological alterations in all the examined organs than other hardness exposed groups. The intestine is the primary route for magnesium (Mg^2^⁺) uptake in fish, while the gills serve as a secondary route. The gill epithelium is selectively permeable and is primarily adapted for the uptake of Na⁺ and Ca^2^⁺ ions, with a lesser affinity for Mg^2^⁺ (Bijvelds et al. 1998). Mg^2^⁺ ions have a larger hydrated radius, which results in the water molecules within their hydration shell being tightly bound. These unique chemical properties suggest that Mg^2^⁺ transport proteins are distinct from those for other ions, such as Na⁺ and Ca^2^⁺ (Moomaw and Maguire 2008). Consequently, the concentration of Mg^2^⁺ in the plasma is typically insufficient to cause severe histopathological damage.

## Conclusion

The present study demonstrated that long-term exposure of Nile tilapia to elevated water hardness led to oxidative stress damage in several vital organs, including the liver, kidney, spleen, and brain. This resulted in significant morphological abnormalities, increased levels of ion content, increased liver enzymes activity and kidney biochemical markers, as well as elevated oxidative stress indicators. The study also demonstrated that CaCl₂ mainly targets the liver and kidney, while NaHCO₃ primarily affects the gills, kidney, and spleen. Both CaCl₂ and NaHCO₃ hardness had a more detrimental impact on Nile tilapia than MgCl₂.

Based on the results of the present work, we advised fish growers to consistently assess water-hardness levels in the tilapia aquaculture systems to mitigate health hazards. Nevertheless, further laboratory and/or field investigations are necessary to determine the mechanistic and ecological implications of major ion toxicity and to create approaches to safeguard aquatic life and guarantee food safety. Additionally, future studies are required to ascertain the long-term effect of individual ions and their mixture on the reproductive performance and immune status of Nile tilapia.

## Supplementary Information

Below is the link to the electronic supplementary material.

Supplementary file1 (DOCX 172 KB)

## Data Availability

All data will be made available on request.
