# Supplementary material for: The effect of long-term exposure to single and mixed mineral ions related to water hardness on Nile tilapia (Oreochromis Niloticus)
Source: Fish Physiol Biochem. 2025 Jul 17;51(4):122. doi: 10.1007/s10695-025-01532-9 (PMC12271268; doi:10.1007/s10695-025-01532-9)
Supplement: Supplementary file 1 — Supplementary file1 (DOCX 172 KB) [file 10695_2025_1532_MOESM1_ESM.docx]

**
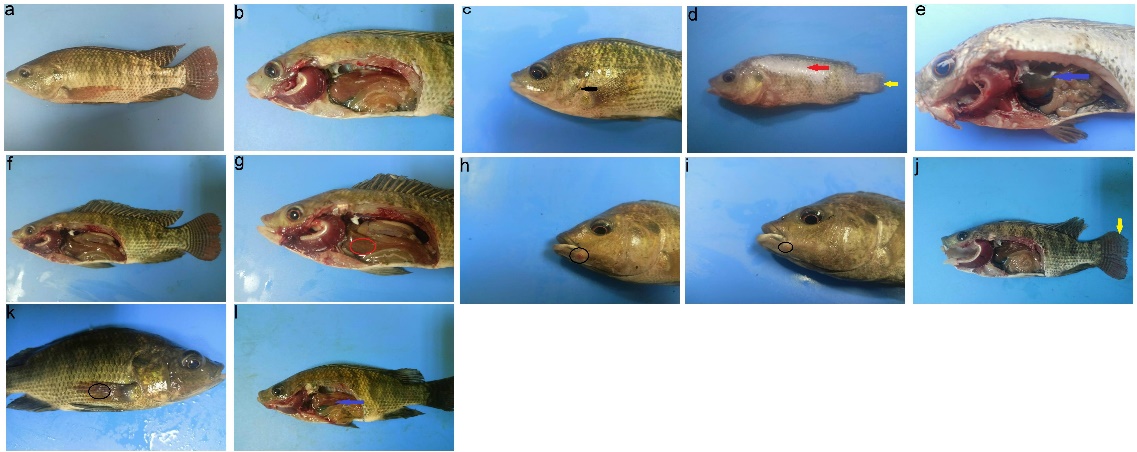
**

**Fig. S1.** The effect of different water hardness treatments on the gross appearance of fish (*O. niloticus).* (a-b) control group; (c-e) Ca group; (f, g) Mg group; (h-j) Na group; (k, l) Mix salts group. Note, (black arrow) black spots on the anterior part of body, (red arrow) loss of scales, (yellow arrow) turned tail fins, (blue arrow) darkness in liver, (red circle) mottled liver, (black circle) vesicles on the lower lip and pectoral fin.
